# (GTG)_5_ MSP-PCR Fingerprinting as a Technique for Discrimination of Wine Associated Yeasts?

**DOI:** 10.1371/journal.pone.0105870

**Published:** 2014-08-29

**Authors:** Mauricio Ramírez-Castrillón, Sandra Denise Camargo Mendes, Mario Inostroza-Ponta, Patricia Valente

**Affiliations:** 1 Centro de Biotecnologia, Universidade Federal do Rio Grande do Sul, Campus do Vale, Porto Alegre, Brazil; 2 Departamento de Microbiologia, Imunologia e Parasitologia, ICBS, Universidade Federal do Rio Grande do Sul, Rua Sarmento Leite, Porto Alegre, Brazil; 3 Empresa de Pesquisa Agropecuária e Extensão Rural de Santa Catarina, Laboratório de Análises de Vinhos e Derivados, Estação Experimental de Videira, Campo Experimental, Videira, Brazil; 4 Departamento de Ingeniería Informática, Universidad de Santiago de Chile, Santiago de Chile, Chile; Louis, University of Leicester,; United Kingdom

## Abstract

In microbiology, identification of all isolates by sequencing is still unfeasible in small research laboratories. Therefore, many yeast diversity studies follow a screening procedure consisting of clustering the yeast isolates using MSP-PCR fingerprinting, followed by identification of one or a few selected representatives of each cluster by sequencing. Although this procedure has been widely applied in the literature, it has not been properly validated. We evaluated a standardized protocol using MSP-PCR fingerprinting with the primers (GTG)_5_ and M13 for the discrimination of wine associated yeasts in South Brazil. Two datasets were used: yeasts isolated from bottled wines and vineyard environments. We compared the discriminatory power of both primers in a subset of 16 strains, choosing the primer (GTG)_5_ for further evaluation. Afterwards, we applied this technique to 245 strains, and compared the results with the identification obtained by partial sequencing of the LSU rRNA gene, considered as the gold standard. An array matrix was constructed for each dataset and used as input for clustering with two methods (hierarchical dendrograms and QAPGrid layout). For both yeast datasets, unrelated species were clustered in the same group. The sensitivity score of (GTG)_5_ MSP-PCR fingerprinting was high, but specificity was low. As a conclusion, the yeast diversity inferred in several previous studies may have been underestimated and some isolates were probably misidentified due to the compliance to this screening procedure.

## Introduction

Yeast identification is currently based on sequencing of domains 1 and 2 (D1/D2) of the LSU rRNA gene and/or the ITS1-5.8S-ITS2 region [1], proposed as a universal barcode for fungi in 2011 [2]. Monitoring the contribution of each species or population, both in industrial microbiology or yeast diversity studies, involves the isolation and analysis of a large number of isolates, which makes the identification of all the isolates by sequencing unfeasible in small research laboratories. In this regard, many molecular techniques have been developed to discriminate between different yeast species. Among them, the Microsatellite/Minisatellite Primed (MSP)-PCR Fingerprinting technique has been widely applied in the literature using primers as (GAC)_5_, (GACA)_4_, (GTG)_5_ and M13. For example, the primer (GTG)_5_ was frequently used to discriminate species of the genus *Saccharomyces* [3]–[8], characterize strains of non-*Saccharomyces* species [9]–[12], analyze yeast diversity [13]–[20], and describe new yeast genus and species [21]–[24]. Most of these studies use MSP-PCR fingerprinting as a preliminary clustering step for the choice of representative strains to be sequenced. Identification is ultimately attained by sequencing, and all the strains grouped in the same cluster of the sequenced one are assumed to belong to the same species. Although this procedure has been widely applied in the literature, it has not been properly validated. Furthermore, some studies have reported difficulties in discriminating species using MSP-PCR fingerprinting with different primers [25]–[29]. In this context, each study reports different DNA amplification protocols, jeopardizing the comparison of genetic profiles, and making it impossible to share genotype databases among laboratories.

A MSP-PCR fingerprinting protocol with (GTG)_5_ primer was useful for the description of yeast population dynamics along the fuel-ethanol fermentation process, and for the identification of the dominant wild strains that could be used as starter strains [7]; however, this primer has not yet been evaluated for monitoring the yeast dynamics in wine production in Brazil. Therefore, the objective of this study was {I} to propose and validate a standardized protocol for the MSP-PCR Fingerprinting technique, and {II} to assess its reliability as a tool for discrimination of different yeast species and clustering of isolates belonging to the same species. This protocol was intended to be applied to wine yeasts, and was evaluated using two datasets: yeasts isolated from bottled wines (thereafter considered a "lower diversity" sample), and yeasts from the winery and vineyard environments ("higher diversity" sample). For the validation of the technique, identification by sequencing was selected as gold standard. We found high intra and inter-specific variability in the fingerprint profiles, with clusters comprising isolates belonging to different species, suggesting a high probability of misidentification when MSP-PCR fingerprinting followed by sequencing of representatives of each profile is applied in yeast diversity studies.

## Results and Discussion

### Yeast identification

From the "lower diversity" group of species (isolated from bottled wines), we obtained the genomic DNA of 102 yeast strains, belonging to 11 species, plus 4 non-identified isolates (Table 1). All the isolates were identified by sequencing the D1/D2 domain of the LSU rRNA gene or the ITS1-5.8S-ITS2 region. The analysis was initially performed with the "lower diversity" group of yeasts, and afterwards expanded to the "higher diversity" group. From the "higher diversity" group (isolated from the winery and vineyard environments, see Methods S1), we obtained 101 isolates belonging to 20 species plus 38 non-identified isolates (Table S1).

**Table 1:** Yeasts species from bottled wines sampled in Rio Grande do Sul and Santa Catarina, South Brazil.

Species	Number of strains	Strain code
*Pichia manshurica* *	36	MRC188, MRC163, MRC143, MRC130, MRC142, MRC140, MRC139, MRC141, MRC128, MRC124, MRC106B, MRC133, MRC189, MRC109, MRC110, MRC112, MRC122, MRC123, MRC125, MRC107, MRC114, MRC115, MRC116B, MRC127, MRC136, MRC111, MRC132, MRC113, MRC134, MRC116A, MRC171, MRC185, MRC126, MRC186, MRC121, MRC131
*Dekkera bruxellensis* *	30	MRC178, MRC180, MRC177, MRC88, MRC172, MRC181, MRC117, MRC120, 66E, 67E, 75E, 59E, 60E, 62E, 65E, 68E, 69E, 70E, MRC80, 73E, 77E, 74E, MRC190, MRC78, MRC79, MRC86, MRC87, MRC182, 22E, 71E
*Zygosaccharomyces bailii* *	14	MRC162, MRC161, MRC137, MRC160, MRC187, MRC144, MRC145, MRC156, MRC105, MRC118, MRC119, MRC146, MRC173, 24E
*Pichia membranifaciens* *	8	MRC152A, MRC153, 16E, MRC184, MRC165, MRC152B, MRC166, MRC168,
*Saccharomyces cerevisiae* *	7	MRC154, 26E, 72E, 20E, 15E, MRC164, 19E
*Torulaspora delbrueckii*	2	MRC183, 17E
*Aureubasidium pullulans*	1	MRC148
*Candida magnoliae*	1	MRC179
*Candida zeylanoides*	1	18E
*Zygosaccharomyces bisporus*	1	MRC158
*Hanseniaspora sp.* **	1	MRC81
Non identified	4	MRC129, MRC147, 23E, 25E
**Total**	106	

* These species were assessed for clustering analysis.

**We considered these species as not identified.

### Standardization and assessment of MSP-PCR Fingerprinting profiles

We made an initial screening of a subset of 16 isolates with the primers M13 and (GTG)_5_ to evaluate the discriminatory power of each primer. Both primers generated discriminative and complex fingerprints, with band sizes ranging from 200 to 2500bp for M13 and 200 to 1800bp for (GTG)_5_. Dendrograms for M13 and (GTG)_5_ primers showed four clusters with a discriminatory power (D) of 0.66 for M13 (Figure S1A), and 0.7 for (GTG)_5_ (Figure S1B). Nevertheless, the dendrogram made with the primer (GTG)_5_ grouped all the four isolates of *Saccharomyces cerevisiae* in the same cluster (Figure S1B). Literature concerning the usefulness of these primers is conflicting. For instance, the primer (GTG)_5_ was recommended to monitor populations of yeasts in ethanol fermentation [7]. Several authors demonstrated that non-*Saccharomyces* species participating in different fermentation processes showed similar profiles with M13 and (GACA)_4_ and greater variability using the primers (GAC)_5_ and (GTG)_5_ [13], [16], [30], [31]. On the other hand, the primer M13 was able to differentiate 16 strains of *S. cerevisiae*, although with different amplification conditions [32]. M13 or both M13 and (GTG)_5_ primers are widely used for assessment of yeast communities [33], and description of new genus, species or genotypes within species [21], [34], although Libkind [28] suggested that the primer M13 is not able to separate fingerprinting profiles in a complex of closely related species because it amplifies more conserved regions of DNA. Thus, as our goal was to discriminate related and unrelated yeast species, both primers had similar discriminatory power with our subset of 16 isolates, and the primer (GTG)_5_ grouped all the isolates of *S. cerevisiae* in the same cluster, we chose primer (GTG)_5_ for further evaluation.

The MSP-PCR Fingerprinting was standardized using the (GTG)_5_ primer with the strain 20E (*S. cerevisiae*). The number of bands in the *S. cerevisiae* 20E profile was similar to other (GTG)_5_ fingerprinting profiles obtained for this species in other studies [7], [35], [36]. The technique proved to be repeatable when tested in two independent PCR reactions with six repetitions using the commercial strain CLIB 2048 (*S. cerevisiae*). Repeatability and reproducibility were also evident when randomly chosen strains were analyzed in independent experiments.

We calculated the concordance between the (GTG)_5_ fingerprinting and sequencing using the kappa index for the “lower” and “higher diversity” datasets, taking into account all the bands obtained from each isolate. The 106 isolates from the "lower diversity" dataset and the 139 isolates from the "higher diversity" dataset showed a kappa index of 0.177 and 0.201, respectively, with a confidence interval of 95% (Table 2). This means that the concordance between the identification by sequencing (gold standard) and by the (GTG)_5_ MSP-PCR fingerprinting was slight for the "lower diversity" and fair for the "higher diversity" dataset [37]. High scores of sensitivity (100%, 97.4%) and low scores of specificity (23.3%, 33.7%) with the (GTG)_5_ MSP-PCR fingerprintings were found for the "lower" and "higher" diversity datasets, respectively (Table 2). High sensitivity scores mean that the number of isolates correctly identified by the MSP-PCR fingerprinting was high, but the low specificity scores mean that there were also many misidentified isolates in comparison with the "gold standard". The low specificity scores may explain the low concordance between the MSP-PCR fingerprinting and the sequencing methods in the present study. The source of the samples seemed not to influence the quality of the results, since isolates sampled from bottled wines ("lower diversity" dataset) and from the vineyard environments ("higher diversity" dataset) resulted in similar kappa indexes, sensitivities and specificities. The (GTG)_5_ MSP-PCR fingerprinting has not been previously evaluated for these parameters.

**Table 2:** Specificity, sensitivity and kappa index of MSP-PCR fingerprinting using the primer (GTG)_5_ in comparison with rDNA sequencing as the gold standard for the two datasets (“lower diversity” and “higher diversity”), using two ranges of band sizes: 200–3500bp or 200–900 bp.

Dataset	Range of band size		MSP-PCR fingerprinting	Gold standard (sequencing)	Kappa index
			(GTG_5_)		
“Lower diversity”	200–3500bp	Specificity	23.30%	100.00%	0.177
		Sensitivity	100.00%	41.70%	
	200–900bp	Specificity	15.20%	100.00%	0.169
		Sensitivity	100.00%	60.30%	
“Higher diversity”	200–3500bp	Specificity	33.70%	97.10%	0.201
		Sensitivity	97.40%	35.60%	
	200–900bp	Specificity	20.20%	95.50%	0.124
		Sensitivity	97.70%	36.40%	

In order to understand the effect of the presence/absence of each band obtained by the (GTG)_5_ MSP-PCR fingerprinting for the clustering of the isolates, the discriminatory power (D) of each band within each species was calculated for the 245 isolates and three reference strains. The D value of the bands for the five most abundant species of each dataset ranged from 0.048 to 1.000 (see Tables S2, S3). Many species had bands with D values around 1.000, meaning that those bands were able to discriminate all the isolates within the species, therefore making the fingerprinting profiles dissimilar among isolates from the same species. Bands with molecular weight lower than 900 bp were consistently present in almost all the isolates of each species, whereas the presence of bands with molecular weight higher than 900 bp was more variable (Figures S2, S3). Many factors may contribute for this variable result, and can indicate an amplification bias. Among these factors are the annealing temperature in the PCR, the purity of DNA, the thermocycler equipment, and the electrophoresis conditions for gel migration [38], which interfere with other fingerprinting techniques as well [39]. Furthermore, (GTG)_5_ MSP-PCR fingerprinting was used in many studies with different protocols [34], , and there is not a consensus in the PCR parameters (annealing temperature within a range of 42–60°C, etc), or electrophoresis conditions (for example, agarose concentration with a range of 1.4–2% w/v). This contributes for the weak reproducibility of the technique among laboratories, and jeopardizes any posterior comparison between the fingerprinting results. In order to rule out any influence from the variable bands higher than 900 bp in our analysis, we recalculated the kappa index, specificity and sensitivity scores using a range of band sizes between 200 and 900 bp. However, the results showed that concordance did not improve (Table 2).

In the present work, the fingerprinting profiles were analyzed based only on the number and size of bands, although band intensity is also considered by some authors. It has been previously suggested that the identification of two or more strains with the same amplification pattern (number and intensity of bands) might indicate clonality of strains from different geographical origins [7]. In our study, isolates of the species *Pichia membranifaciens* gave repeatedly the same band patterns without differences due to missing bands, although differences in band intensity of some fingerprints occurred. Therefore, the band intensity was not used as a variable for grouping the isolates in our study.

### The “lower diversity” yeast dataset

When analyzing the "lower diversity" group of yeasts (isolated from bottled wines), and considering only the species with more than 7 isolates, we found DNA fragments of 200 to 3500 bp, with banding patterns containing between 4 and 11 visualized bands (Figure S4). In this dataset, strains identified as *Pichia manshurica, S. cerevisiae*, *Zygosaccharomyces bailii* and *Dekkera bruxellensis* presented different band patterns within each species. For example, some strains of *D. bruxellensis* showed higher bands (approximately 2500 pb) that were absent in others. To exclude error in the PCR reaction as the explanation, the experiment was repeated three times being obtained the same pattern of amplification. This result separated this species into at least two clusters, which might be due to variation in DNA quality (although some nucleic acids were extracted again, quality problem cannot be discarded) or intraspecific variability. In fact, intraspecific variability in *S. cerevisiae* was found using MSP-PCR Fingerprinting with the (GTG)_5_ primer [8]. We found that an increase in the number of isolates raised the number of different band patterns within each species.

**Figure 1:**
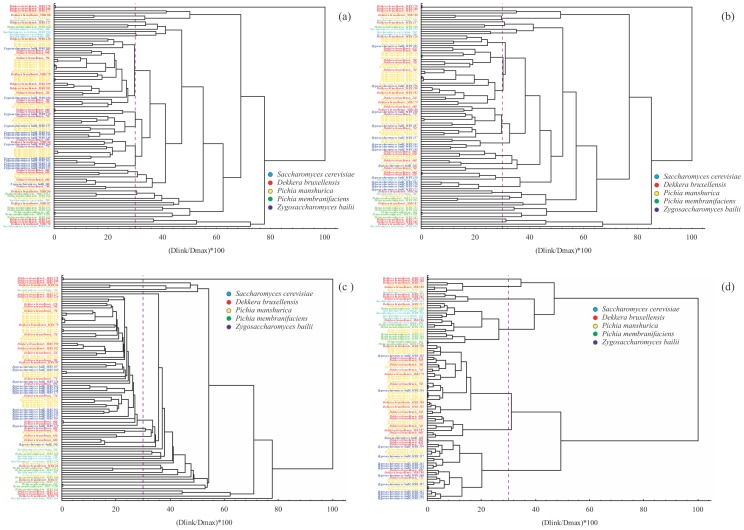
Dendrograms of the clustering of the strains from the "lower diversity" dataset by Hierarchical Clustering using: (a) average linkage (b) complete linkage, (c) single linkage, and (d) Ward´s method. The distance was computed using the Euclidean distance between the genetic profiles based on the MSP-PCR fingerprinting with the primer GTG_5_.

Two clustering strategies, using quantitative data based on the molecular weight of the bands and Euclidean distances, were used to attempt clustering the genotypic profiles obtained with the MSP-PCR fingerprinting technique. First, a Hierarchical Clustering algorithm (with four methods of pairwise analysis) showed dendrograms where the isolates did not group into well-defined clusters (Figure 1). For example, dendrograms with UPGMA, single linkage or complete linkage showed high variability in the clusters with a cut-off of 30%, generating approximately 30 groups with mixed species. The Ward´s method, by contrast, grouped the isolates into seven clusters using the same cut-off, but increased the likelihood of grouping isolates from different species (Figure 1). The other strategy was to use QAPGrid, an unsupervised graph clustering algorithm combined with a combinatorial optimization layout method [46]. As a result, the algorithm found 14 clusters, with the smallest cluster containing two isolates and the largest one containing 15 strains (Figure S2).

In general, the most abundant species were placed in several clusters, and 85% of the clusters were represented by two or more species in the QAPgrid output. As this algorithm groups similar band patterns, unrelated species with similar profiles were joined in the same group, therefore, being the resolution of the clustering poor. For example, we expected to find mixed clusters with the species *P. manshurica* and *P. membranifaciens* because they are sibling species that comprise a species complex [9], but we also found mixed clusters for *D. bruxellensis, Z. bailii* and *S. cerevisiae*, due to the similar genetic profiles (number and size of bands) of some isolates. As the Hierarchical Clustering and the QAPGrid were not capable of grouping the species, we confirmed that the problem was the raw data (fingerprinting patterns) used to construct the matrix analyzed by both methods.

Many yeast diversity studies apply the MSP-PCR fingerprinting to select one or two representative isolates from each pattern for sequencing aiming the identification at the taxonomic level of species [47]–[50]. Based on our results, it might be inferred that yeast diversity was underestimated and some isolates were misidentified in many previous works. In an attempt to assess the probability of misidentification and consequent underestimation of the species richness, we selected two mixed species clusters (with three and 15 isolates, respectively) from the QAPgrid output (Figure S2). For the smaller cluster, two isolates were identified as *P. manshurica* and the other isolate as *D. bruxellensis*. If we select only two isolates from this cluster for sequencing, the probability of misidentification is 33%. The largest cluster contained four different species and, if two representative isolates were selected for sequencing, the probability of misidentification could reach 40%. This illustrates the problem of using the MSP-PCR Fingerprinting with the primer (GTG)_5_ as a technique for grouping isolates in order to select some of them for sequencing.

## Materials and Methods

### Strains and growth conditions

The yeast strains isolated in this study are listed in Table 1 and Table S1. Two sets of samples were included. The first group of yeasts (n = 106) was isolated from South Brazilian bottled wines ("lower diversity" dataset, Table 1), and the second group (n = 139) was isolated from environments surrounding the wineries (vineyard soil, effluent, leaves, fruits, cellars – "higher diversity" dataset, Table S1). Details concerning the isolation of yeast strains can be seen in Methods S1. Field collections were conducted according to EPAGRI diversity rules, and all necessary permits were obtained for the field studies (Codes 1414, 13214). Reference strains used in this study were: *Saccharomyces cerevisiae* CLIB 2048, *Saccharomyces bayanus* CLIB 2033 and *Saccharomyces uvarum* CLIB 2028 (Collection de Levures d’Interet Biotechnologique, Paris-Grignon, France).

### DNA extraction

Two protocols were used in this study. DNA of yeasts isolated from bottled wine was extracted with the potassium acetate-based protocol proposed by [51] with some modifications. Pure colonies of each strain were grown in GYP broth at 30°C for 18 hours. After centrifugation and washing with distilled water, the biomass of each culture was re-suspended in 400µL of lysis buffer (0.5 M NaCl, 10 mM EDTA, 2% SDS, 50 mM Tris-HCl, pH 8) and incubated for 60 min at 65°C. The other steps were done as described in [51]. Genomic DNA of samples collected in the second group was extracted using the classic protocol with phenol/chloroform [52]. The quality of the extracted DNA was analyzed on agarose gels (1% w/v) and assessing the A260/A280 ratio.

### MSP-PCR Fingerprinting

MSP-PCR Fingerprinting using the primers (GTG)_5_ or M13 was optimized from [7] using strain 20E (*S. cerevisiae*). Different concentrations of each reagent used in PCR were tested: MgCl_2_ (1.5–4.5 mM), primer (0.2–1.4 pmol/μL), dNTPs (10–70µM) and DNA (110–0.1 ng/μL). The optimized reaction mix for a volume of 25µL was: 1 U of Taq polimerase (Invitrogen), 1X buffer reaction, 3 mM MgCl_2_, 1 pmol/μL primer, 60µM dNTPs Mix and 5µL of DNA (1 ng/μL). The program started at 94°C for 5 min followed by 35 cycles at 94°C for 15 s, 55°C for 45 s, and 72°C for 90 s, with final extension at 72°C for 6 min.

The PCR products were separated in 1.8% (w/v) agarose gels (Bioron, Ludwigshafen, Germany; 12.5 cm width; 8.5 cm height) made in 1X TAE buffer (40 mM Tris–Acetate, 1 mM EDTA, pH 8.0) using electrophoresis with stacking: initial migration at 110 V for 5 min followed by 70 V for 180 min. Gels were stained with GelRed (Biotium, Hayward, USA) for visualization under UV light and digital image capturing was done using the Geni2 gelDoc System (Syngene, Cambridge, UK). The resulting fingerprints were analyzed using the software GeneTools. The 1 Kb plus or 1 Kb (Invitrogen) molecular weight marker was used to compare the sizes of the bands.

### Yeast molecular identification

The divergent D1/D2 domain of the LSU rRNA gene was amplified and sequenced with NL1 and NL4 primers [53]. The ITS1-5.8S-ITS2 region was amplified and sequenced with ITS1 and ITS4 primers [54]. Amplification conditions were as follows: one initial cycle at 94°C for 5 min, 35 cycles at 94°C for 15 s, 55°C for 45 s, 72°C for 90 s, and a final extension cycle at 72°C for 6 min. The PCR products were examined by electrophoresis on a 1.5% agarose gel at 100 V for 45 min and stained with GelRed for visualization under UV light. Digital image capturing was done using the Geni2 gelDoc System (Syngene, Cambridge, UK).

The sequences were obtained with ABI-PRISM 3100 Genetic Analyzer (Life Technologies Corp., USA) using standard protocols at the “Ludwig Biotecnologia” facility in Alvorada-RS, Brazil, and were compared with the sequences of type strains published in the GenBank database using the software YeastIP [55]. A cut-off of 99% similarity was used to identify the isolates.

### Clustering analysis

Two clustering algorithms were used to group the (GTG)_5_ MSP-PCR Fingerprinting profiles: (a) a Hierarchical Clustering algorithm with four versions for pairwise analysis: average linkage, complete linkage, single linkage and Ward´s method; (b) QAPGrid, an unsupervised graph clustering algorithm combined with a combinatorial optimization layout method [46].

For both clustering algorithms, a matrix was constructed considering each isolate and the total number of bands (n = 23), with the size of each band for each isolate. The size of the bands took into consideration a deviation of 50 bp for the smallest bands, and 200 bp for the largest ones, due to the agarose gel resolution. Thus, each isolate was represented by 23 integer numbers corresponding to the size of the bands found by the MSP-PCR Fingerprinting method. If a band were not present for an isolate, we considered a value of zero for that band. We used a Euclidean distance between each pair of isolates to compute the distance of the genetic profiles of isolates. The matrix is available in Dataset S1.

The second method incorporates the use of a graph-based clustering algorithm that automatically finds the number of clusters based on the distance between the genetic profiles of the isolates. After the clustering is performed, the QAPGrid algorithm produces a layout representative of the clusters. Details of the clustering and layout algorithms can be found in Inostroza-Ponta et al. [46], [56]. This combination has been successfully applied in the analysis of other type of genetic data [57]–[58].

### Discriminatory power

In order to compare the discriminatory power (D) of the primers M13 and (GTG)_5_ in the MSP-PCR fingerprinting, we used the index of discrimination proposed by [59]–[60], which is based on the Simpson's index of diversity. The discriminatory power was calculated based on a subset of 16 strains from the "lower diversity" dataset. Dendrograms were constructed based on the Ward´s method and Euclidean distances, and grouped with a cut-off of 50%.

The equation used for the calculation of the discriminatory power is as follows:



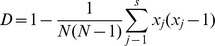



where D is the index of discriminatory power, N, the number of unrelated strains tested, S, the number of different types, and x_j_, the number of strains belonging to the j^th^ type, assuming that strains will be classified into mutually exclusive categories. A D value of 1.0 indicates that the primer was able to distinguish each isolate of a community from all other members of that community. Conversely, an index of 0.0 indicates that all isolates of a community were of an identical type [59]–[60].

Afterwards, the discriminatory power of each band of the MSP-PCR fingerprinting profile with the primer (GTG)_5_ was calculated for 208 isolates and the three reference strains according to the equation described above. The discriminatory power (D) of each band obtained in the (GTG)_5_ MSP-PCR fingerprinting was calculated as the measurement of the variation of “alleles” (presence or absence of bands) by each “locus” (band position), with a range between zero (homogeneity) and one (heterogeneity). A low D indicates a “locus” with similar "alleles" (presence or absence of bands), while a high D indicates a “locus” with an irregular presence of bands among the isolates.

### Concordance between the (GTG)_5_ MSP-PCR fingerprinting and sequencing, sensitivity and specificity assessments

We evaluated the concordance between the identification by (GTG)_5_ MSP-PCR fingerprinting and by sequencing using the whole “lower diversity” (n = 106) and “higher diversity” (n = 139) datasets, and the Kappa index [61]. The sensitivity and specificity indexes were assessed using the McNemar test for comparison of the results obtained by sequencing (considered as the gold standard) and the ones obtained by the (GTG)_5_ MSP-PCR fingerprinting [62]. The sensitivity indicates the percentage of isolates identified by sequencing that were identified as the same species by the MSP-PCR fingerprinting (true positive isolates), and is a measure of the probability that an isolate belonging to a certain species will be correctly identified at that species by the (GTG)_5_ MSP-PCR fingerprinting. The specificity indicates the percentage of isolates that were not identified in a certain species by the sequencing methodology which were not identified in that species by the MSP-PCR fingerprinting (true negatives) either. We considered the isolates not identified by sequencing as true negative results. All the tests were estimated with a confidence interval of 95%.

## Supporting Information

Figure S1 **Dendrograms of the MSP-PCR fingerprinting profiles with the primers M13 (a) and (GTG)_5_ (b) of a subset of 16 strains from the "lower diversity" dataset for the analysis of the discriminatory power of the primers.** The dendrograms were constructed by the Hierarchical Clustering using the Ward´s method, and the distance was computed using the Euclidean distance between the genetic profiles. We used a cut-off of 50% for the calculation of the discriminatory power.
(TIF)

Figure S2 **QAPGrid layout for the clustering of the strains from the "lower diversity" dataset.** The distance was computed using the Euclidean distance between the genetic profiles based on the MSP-PCR fingerprinting with the primer GTG_5_. Each strain is represented as a bar chart. The colors represent the different species based on the molecular identification, and the legend is the same as in Figs. 1A and 1B. Each bar represents one band in the fingerprinting profile of each strain, the horizontal axis shows the band position in the fingerprinting, and the vertical axis represents the size of the band (bp). The dashed lines indicate the two clusters (smaller and bigger) used for the calculation of the probability of misidentification and consequent underestimation of the species richness.
(TIF)

Figure S3 **Layout of the MSP-PCR fingerprinting profiles with the primer (GTG)_5_ of the most abundant species within the "higher diversity" dataset.** Each symbol represents one band in the fingerprinting profile. *S. cerevisiae* (a), *H. uvarum* (b), *P. kudriavzevii* (c) and *P. occidentalis* (d). The profiles of the reference strains *S. bayanus* CLIB 2033 *S. uvarum* CLIB 2028 and *S. cerevisiae* CLIB 2048 are shown in Fig. S2a. Each symbol represents one band in the fingerprinting profile of each strain, and the vertical axis shows the size of the band (bp).
(TIF)

Figure S4 **Representative agarose gel of MSP-PCR fingerprinting using the primer (GTG)_5_.** 01: *Dekkera bruxellensis* MRC181; 02: *Pichia manshurica* MRC163; 03: *D. bruxellensis* MRC172; 04: *Pichia membranifaciens* MRC152A; 05: *D. bruxellensis* MRC177; 06: *Torulaspora delbrueckii* MRC183; 07: *Zygosaccharomyces bailii* MRC162; 08: *D. bruxellensis* MRC178; 10: *D. bruxellensis* MRC180; 11: *D. bruxellensis* MRC88; 12: *P. manshurica* MRC188. 1Kb Plus was used as Molecular Weight Marker (MPM).
(TIF)

Table S1 **Yeast species from the vineyard and winery environments collected in Santa Catarina, Brazil.**
(DOC)

Table S2 **Discriminatory power of each band obtained by the MSP-PCR fingerprinting with (GTG)_5_ for the five most abundant species from the "lower diversity" dataset.**
(DOC)

Table S3 **Discriminatory power of each band obtained by the MSP-PCR fingerprinting with (GTG)_5_ for the five most abundant species from the "higher diversity" dataset.**
(DOC)

Methods S1 **Detailed methods for yeast isolation experiments.**
(DOC)

Dataset S1 **Data matrix.**
(XLS)
