# Supplementary figures and images for: (GTG)5 MSP-PCR Fingerprinting as a Technique for Discrimination of Wine Associated Yeasts?
Source: PLoS One. 2014 Aug 29;9(8):e105870. doi: 10.1371/journal.pone.0105870 (PMC4149466; doi:10.1371/journal.pone.0105870)

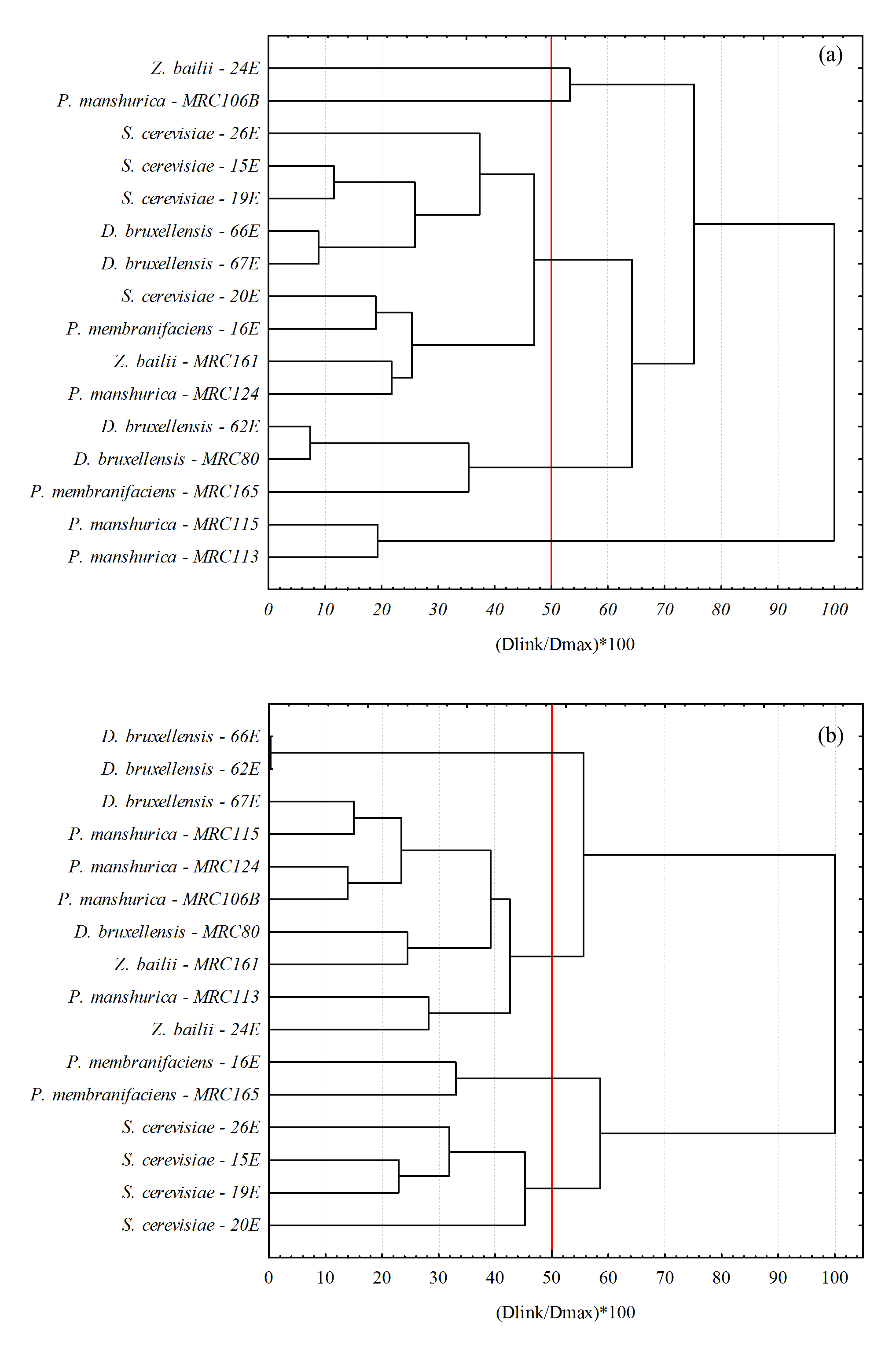

Supplement: Figure S1 — Dendrograms of the MSP-PCR fingerprinting profiles with the primers M13 (a) and (GTG)5 (b) of a subset of 16 strains from the "lower diversity" dataset for the analysis of the discriminatory power of the primers. The dendrograms were constructed by the Hierarchical Clustering using the Ward´s method, and the distance was computed using the Euclidean distance between the genetic profiles. We used a cut-off of 50% for the calculation of the discriminatory power. (TIF) [file pone.0105870.s001.tif]

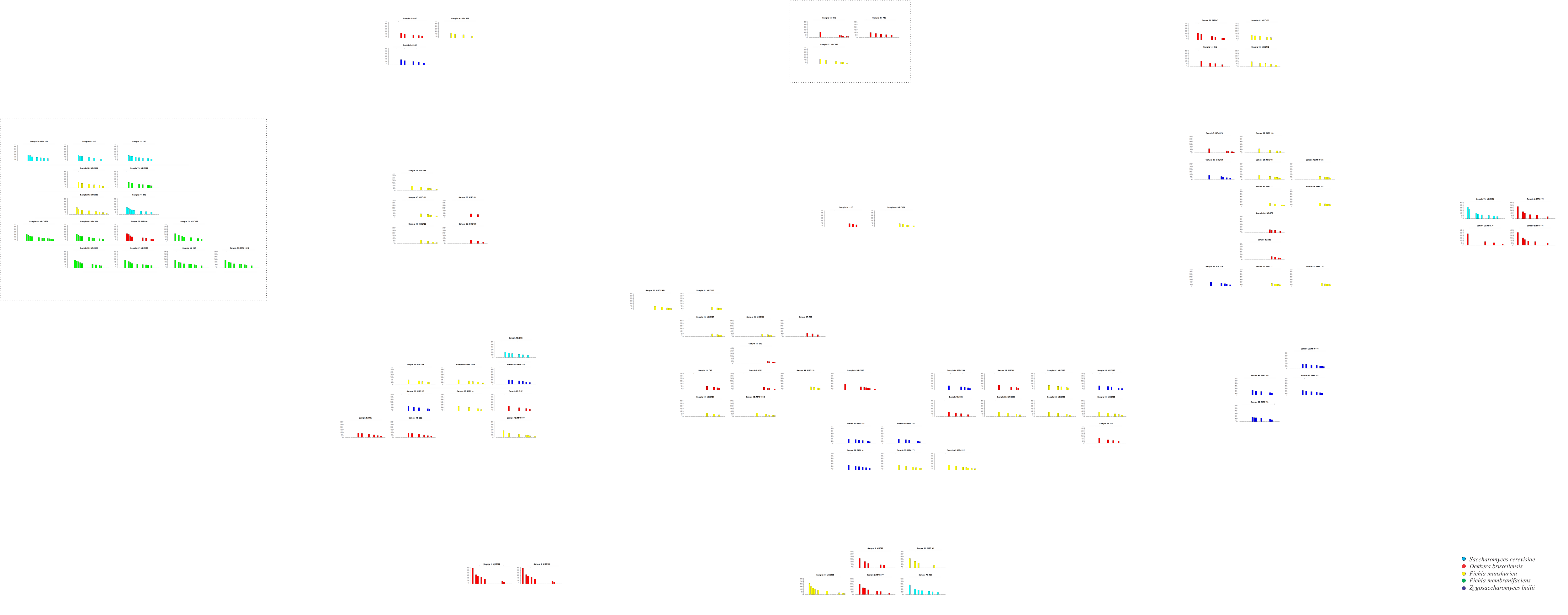

Supplement: Figure S2 — QAPGrid layout for the clustering of the strains from the "lower diversity" dataset. The distance was computed using the Euclidean distance between the genetic profiles based on the MSP-PCR fingerprinting with the primer GTG5. Each strain is represented as a bar chart. The colors represent the different species based on the molecular identification, and the legend is the same as in Figs. 1A and 1B. Each bar represents one band in the fingerprinting profile of each strain, the horizontal axis shows the band position in the fingerprinting, and the vertical axis represents the size of the band (bp). The dashed lines indicate the two clusters (smaller and bigger) used for the calculation of the probability of misidentification and consequent underestimation of the species richness. (TIF) [file pone.0105870.s002.tif]

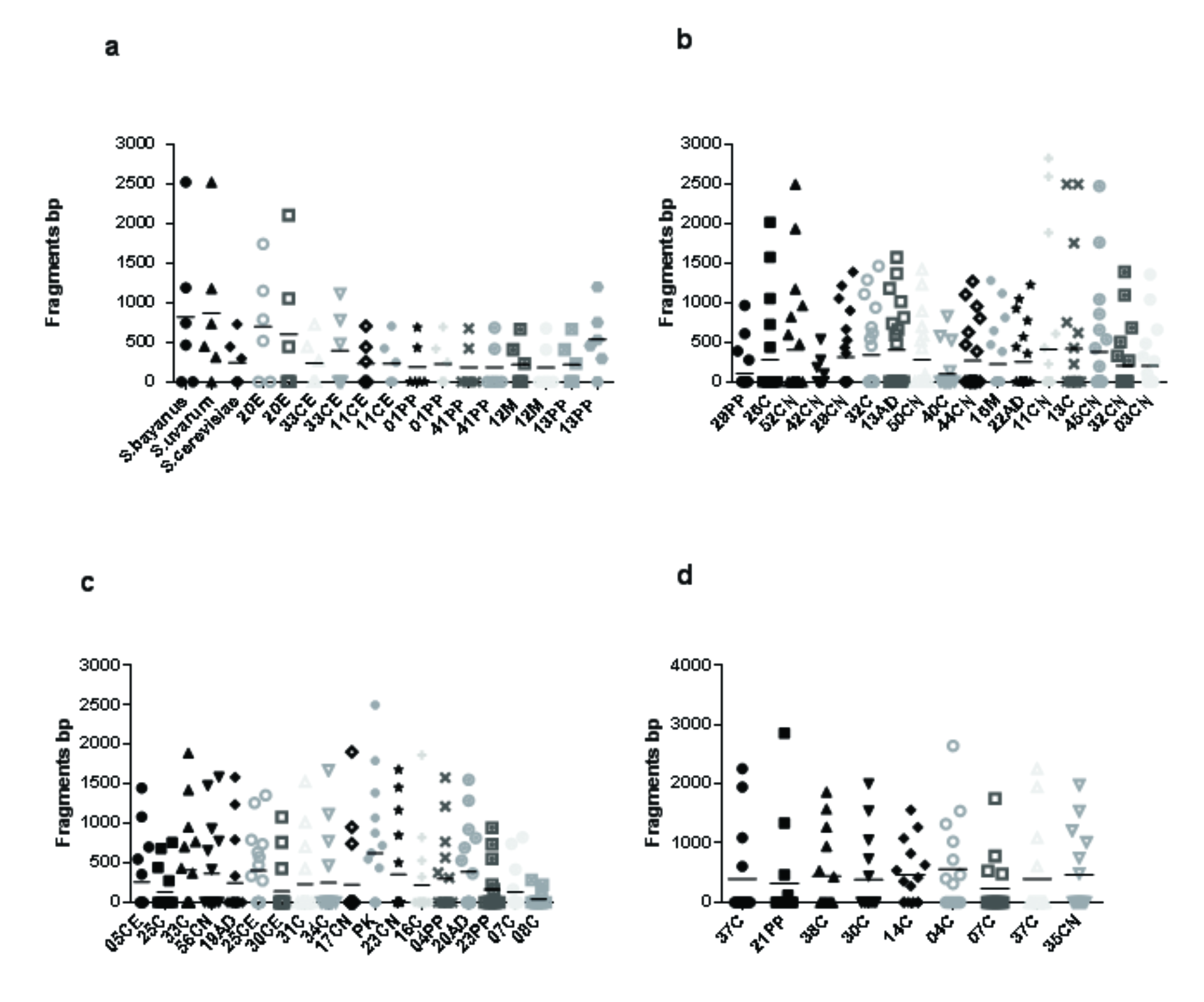

Supplement: Figure S3 — Layout of the MSP-PCR fingerprinting profiles with the primer (GTG)5 of the most abundant species within the "higher diversity" dataset. Each symbol represents one band in the fingerprinting profile. S. cerevisiae (a), H. uvarum (b), P. kudriavzevii (c) and P. occidentalis (d). The profiles of the reference strains S. bayanus CLIB 2033 S. uvarum CLIB 2028 and S. cerevisiae CLIB 2048 are shown in Fig. S2a. Each symbol represents one band in the fingerprinting profile of each strain, and the vertical axis shows the size of the band (bp). (TIF) [file pone.0105870.s003.tif]

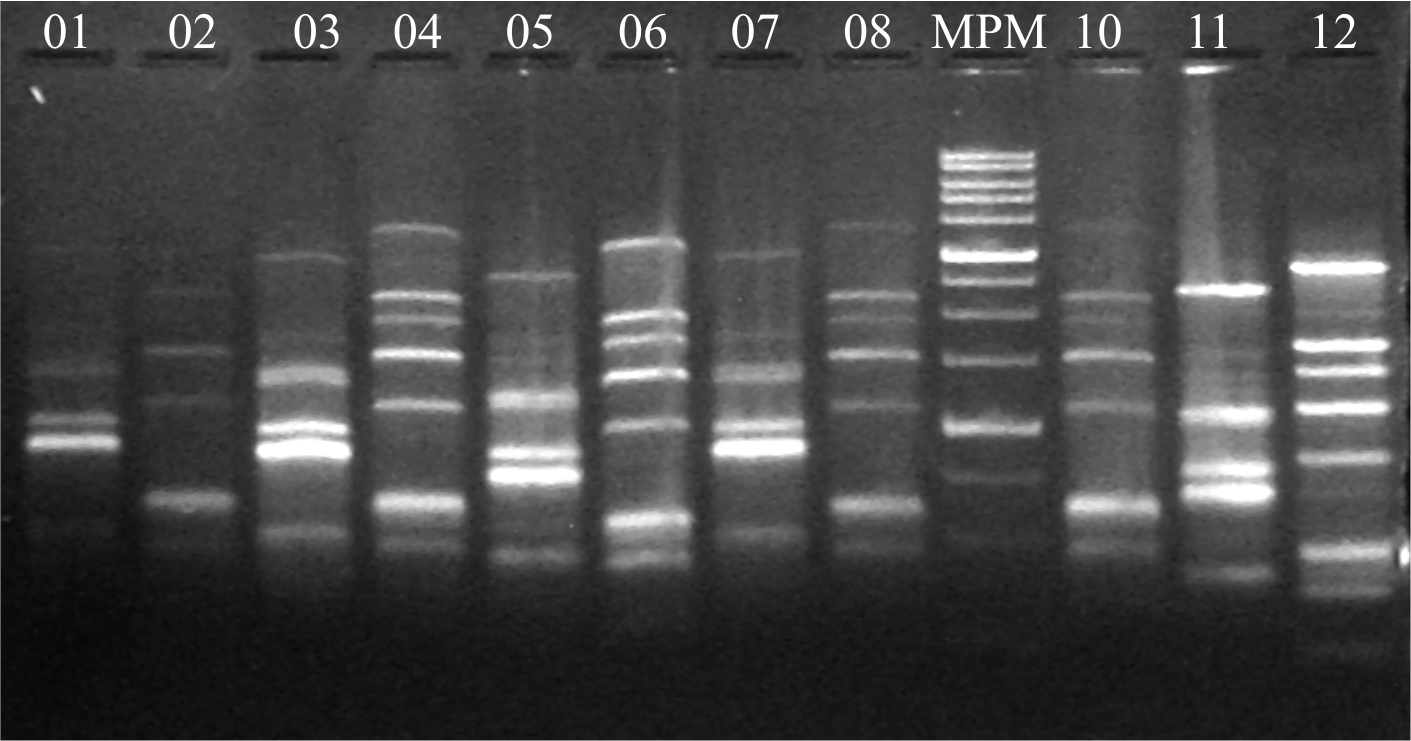

Supplement: Figure S4 — Representative agarose gel of MSP-PCR fingerprinting using the primer (GTG)5. 01: Dekkera bruxellensis MRC181; 02: Pichia manshurica MRC163; 03: D. bruxellensis MRC172; 04: Pichia membranifaciens MRC152A; 05: D. bruxellensis MRC177; 06: Torulaspora delbrueckii MRC183; 07: Zygosaccharomyces bailii MRC162; 08: D. bruxellensis MRC178; 10: D. bruxellensis MRC180; 11: D. bruxellensis MRC88; 12: P. manshurica MRC188. 1Kb Plus was used as Molecular Weight Marker (MPM). (TIF) [file pone.0105870.s004.tif]
