# Supplementary material for: (GTG)5 MSP-PCR Fingerprinting as a Technique for Discrimination of Wine Associated Yeasts?
Source: PLoS One. 2014 Aug 29;9(8):e105870. doi: 10.1371/journal.pone.0105870 (PMC4149466; doi:10.1371/journal.pone.0105870)
Supplement: Table S1 — Yeast species from the vineyard and winery environments collected in Santa Catarina, Brazil. (DOC) [file pone.0105870.s005.doc]

**Table S1.**Yeast species from the vineyard and winery environments collected in Santa Catarina, Brazil.

| **Species** | **Number of strains** |
| --- | --- |
| *Hanseniaspora uvarum* | *32* |
| *Pichia kudriavzevii* | *19* |
| *Pichia occidentalis* | *11* |
| *Saccharomyces cerevisiae* | *9* |
| *Meyerozyma guilliermondii* | *5* |
| *Pichia manshurica* | *3* |
| *Meyerozyma caribbica* | *3* |
| *Hanseniaspora opuntiae* | *3* |
| *Metschnikowia chrysoperlae* | *2* |
| *Clavispora lusitaniae* | *2* |
| *Hanseniaspora clermontiae* | *2* |
| *Candida zemplinina* | *2* |
| *Candida allociferri* | *1* |
| *Trichomonascus ciferri* | *1* |
| *Rhodotorula mucilaginosa* | *1* |
| *Metschnikowia pulcherrima* | *1* |
| *Zygoascus meyerae* | *1* |
| *Candida intermedia* | *1* |
| *Pichia membranifaciens* | *1* |
| *Pichia aff fermentans* | *1* |
| Non identified | *38* |
| **Total** | *139* |
