# Supplementary material for: (GTG)5 MSP-PCR Fingerprinting as a Technique for Discrimination of Wine Associated Yeasts?
Source: PLoS One. 2014 Aug 29;9(8):e105870. doi: 10.1371/journal.pone.0105870 (PMC4149466; doi:10.1371/journal.pone.0105870)
Supplement: Table S2 — Discriminatory power of each band obtained by the MSP-PCR fingerprinting with (GTG)5 for the five most abundant species from the "lower diversity" dataset. (DOC) [file pone.0105870.s006.doc]

**Table S2.** Discriminatory power of each band obtained by the MSP-PCR fingerprinting with (GTG)_5_ for the five most abundant species from the "lower diversity" dataset.

| **Species** |  | **Discriminatory Index (D)** | |  |
| --- | --- | --- | --- | --- |
|  | **Number of Strains** | **D Value of bands** | **C. Interval^1^** | |
| *D. bruxellensis* | 30 | 0.065-1.000 | 0.181-1.000 | |
| *P. manshurica* | 36 | 0.053-0.973 | 0.151-0.950 | |
| *P. membranifaciens* | 9 | 0.180-0.880 | 0.478-0.930 | |
| *S. cerevisiae* | 7 | 0.118-1.000 | 0.308-0.950 | |
| *Z. bailii* | 14 | 0.124-0.933 | 0.343-0.950 | |

^1^ Confidence Interval = Precision of the Discriminatory Index, expressed as 95% upper and lower boundaries.
