# Supplementary material for: (GTG)5 MSP-PCR Fingerprinting as a Technique for Discrimination of Wine Associated Yeasts?
Source: PLoS One. 2014 Aug 29;9(8):e105870. doi: 10.1371/journal.pone.0105870 (PMC4149466; doi:10.1371/journal.pone.0105870)
Supplement: Table S3 — Discriminatory power of each band obtained by the MSP-PCR fingerprinting with (GTG)5 for the five most abundant species from the "higher diversity" dataset. (DOC) [file pone.0105870.s007.doc]

**Table S3.** Discriminatory power of each band obtained by the MSP-PCR fingerprinting with (GTG)_5_ for the five most abundant species from the "higher diversity" dataset.

| **Species** |  | **Discriminatory Index (D)** | |
| --- | --- | --- | --- |
|  | **Number of Strains** | **D Value of bands** | **C. Interval^1^** |
| *H. uvarum* | 32 | 0.048-1.000 | 0.135-1.000 |
| *P. kudriavzevii* | 19 | 0.080-1.000 | 0.221-1.000 |
| *P. occidentalis* | 11 | 0.222-1.000 | 0.542-1.000 |
| *S. cerevisiae* | 9 | 0.118-1.000 | 0.308-0.950 |
| *M. guilliermondii* | 5 | 0.250-0.964 | 0.594-1.000 |

^1^ Confidence Interval = Precision of the Discriminatory Index, expressed as 95% upper and lower boundaries.
