# Supplementary material for: (GTG)5 MSP-PCR Fingerprinting as a Technique for Discrimination of Wine Associated Yeasts?
Source: PLoS One. 2014 Aug 29;9(8):e105870. doi: 10.1371/journal.pone.0105870 (PMC4149466; doi:10.1371/journal.pone.0105870)
Supplement: Methods S1 — Detailed methods for yeast isolation experiments. (DOC) [file pone.0105870.s008.doc]

**Supplementary Methods**

**Yeast isolation in the "lower diversity" dataset**

Fine and colonial, white and red bottled wine samples (n=49) were collected directly at the point of wine production and distribution, in the states of “Rio Grande do Sul” and “Santa Catarina” (Brazil), between 2003 and 2011. From each bottle, 100mL of pure wine and dilutions in sterile distilled water (1:1, 1:3) were filtered in 45µm porous membranes, and grown in GYP agar plates (2% glucose, 1% peptone, 0.5% yeast extract, 2% agar) for 72 hours at 28ºC. Strains representing the different morphotypes were isolated and purified in GYP medium and / or cultured for molecular analysis.

**Yeast isolation in the “higher diversity” dataset**

The three sampled areas were located in the middle-west of Santa Catarina state, Brazil: Pinheiro Preto (latitude 7 °19’83”S, longitude: 50°49’18”W, height 698 m), Campos Novos (latitude 7º19’83”S, longitude 50°49’18”W, height 973 m), and Marari (latitude 27°12’24”S, longitude 51°06’96”W, height 1053 m). Each area belonged to a vineyard (this area not involve endangered or protected species) and had 10 points sampled in duplicate for two consecutive years (2010-2011). We sampled leaves, healthy and damaged grape curls, the effluent, and the cellar equipment from each vineyard, and the samples were processed as indicated by [1-3] for the isolation of the yeasts.

**References**

1. Prakitchaiwattana CJ, Fleet GH, Heard GM (2004) Application and evaluation of denaturing gradient gel electrophoresis to analyze the yeast ecology of wine grapes. *FEMS Yeast Res* 4:865–877.
2. Kurtzman CP, Fell JW, Boekhout T (2011) TheYeasts, a taxonomic Study, 5^th^ edn., Amsterdam: Elsevier.
3. A.P.H.A. (1992) Standard Methods for the Examination of Water and Wastewater, 14th Edition. American Public Health Association, Washington D.C., P 922-926.
